# Thrombopoietin receptor agonist antibody for treating chemotherapy-induced thrombocytopenia

**DOI:** 10.1186/s12885-023-10975-3

**Published:** 2023-05-31

**Authors:** Jiwon Shin, Min-Jung Kim, Xingguo Quan, Ji Woong Kim, Sukmook Lee, SaeGwang Park, Jee-Yeong Jeong, Kyungmoo Yea

**Affiliations:** 1 Department of New Biology, Daegu Gyeongbuk Institute of Science and Technology (DGIST), Daegu, 42988 Republic of Korea; 2 Department of Biochemistry, Kosin University College of Medicine, Busan, 49267 Republic of Korea; 3 Department of Microbiology and Immunology, Inje University College of Medicine, Busan, 47392 Republic of Korea; 4 Department of Biochemistry, Kookmin University, Seoul, 02707 Republic of Korea; 5 New Biology Research Center, DGIST, Daegu, 43024 Republic of Korea

**Keywords:** Chemotherapy-induced thrombocytopenia, Thrombopoietin receptor, Agonist antibody, Megakaryopoiesis, Platelet production

## Abstract

**Background:**

Thrombocytopenia is a common complication in cancer patients undergoing chemotherapy. Chemotherapy-induced thrombocytopenia (CIT) leads to dose reduction and treatment delays, lowering chemotherapy efficacy and survival rate. Thus, rapid recovery and continuous maintenance of platelet count during chemotherapy cycles are crucial in patients with CIT. Thrombopoietin (TPO) and its receptor, myeloid proliferative leukemia (MPL) protein, play a major role in platelet production. Although several MPL agonists have been developed to regulate thrombopoiesis, none have been approved for the management of CIT due to concerns regarding efficacy or safety. Therefore, the development of effective MPL agonists for treating CIT needs to be further expanded.

**Methods:**

Anti-MPL antibodies were selected from the human combinatorial antibody phage libraries using phage display. We identified 2R13 as the most active clone among the binding antibodies via cell proliferation assay using BaF3/MPL cells. The effect of 2R13 on megakaryocyte differentiation was evaluated in peripheral blood CD34^+^ cells by analyzing megakaryocyte-specific differentiation markers (CD41a^+^ and CD42b^+^) and DNA ploidy using flow cytometry. The 2R13-induced platelet production was examined in 8- to 10-week-old wild-type BALB/c female mice and a thrombocytopenia mouse model established by intraperitoneal injection of 5-fluorouracil (150 mg/kg). The platelet counts were monitored twice a week over 14 days post-initiation of treatment with a single injection of 2R13, or recombinant human TPO (rhTPO) for seven consecutive days.

**Results:**

We found that 2R13 specifically interacted with MPL and activated its signaling pathways. 2R13 stimulated megakaryocyte differentiation, evidenced by increasing the proportion of high-ploidy (≥ 8N) megakaryocytes in peripheral blood-CD34^+^ cells. The platelet count was increased by a single injection of 2R13 for up to 14 days. Injection of 5-fluorouracil considerably reduced the platelet count by day 4, which was recovered by 2R13. The platelets produced by 2R13 sustained a higher count than that achieved using seven consecutive injections of rhTPO.

**Conclusions:**

Our findings suggest that 2R13 is a promising therapeutic agent for CIT treatment.

**Supplementary Information:**

The online version contains supplementary material available at 10.1186/s12885-023-10975-3.

## Background

Thrombocytopenia frequently occurs in patients with cancer because of chemotherapy, the malignancy itself, or infection [1]. Chemotherapy-induced thrombocytopenia (CIT, platelet count ≤ 100,000/μL after chemotherapy), occurs in approximately 16%–30% of patients receiving platinum-, taxane-, or gemcitabine-based regimens [2, 3]. However, no CIT management agents have received approval from the United States Food and Drug Administration (FDA). CIT is associated with hematologic toxicity caused by hematopoiesis suppression, a significant adverse effect of chemotherapy. Since bleeding events are fatal in patients with CIT, surgical procedures, radio-, and chemotherapy are cautiously administered [4]. Therefore, the current standard management for CIT involves the postponement of chemotherapy cycles and decreasing doses to restore the platelet count to the desired level for subsequent treatments. Unfortunately, such treatment approaches lead to reduced relative dose intensity (RDI), which in turn substantially lowers the efficacy of chemotherapy and the survival rate of patients [5].

Thrombopoiesis is regulated at multiple levels by various cytokines, the most important of which is thrombopoietin (TPO). TPO and its receptor, myeloid proliferative leukemia (MPL) protein, govern the megakaryocytic lineage from hematopoietic stem cells and stimulate megakaryocyte (MK) maturation in the bone marrow (BM) niche, thereby promoting platelet production [6]. Recombinant human TPO (rhTPO) and pegylated rhTPO have been extensively studied in patients with CIT. Unfortunately, their therapeutic application ceased owing to the generation of neutralizing antibodies that cross-reacted with endogenous TPO, causing severe thrombocytopenia [7, 8]. Subsequently, the development of alternative MPL agonists has revived interest in CIT treatment. Eltrombopag and avatrombopag are oral small-molecule MPL agonists, whereas romiplostim is a subcutaneously administered peptibody. These agonists have no sequence homology with endogenous TPO, and no anti-drug antibodies have been reported [9]. Clinical studies show that small-molecule MPL agonists were less potent at increasing the platelet count than maximal doses of romiplostim in healthy subjects [10–13]. Moreover, avatrombopag failed to show efficacy in treating CIT, with insignificant results under clinical trials [14]. Although there is increasing evidence to indicate that romiplostim may be effective in CIT management, the evaluation of the safety and efficacy of romiplostim use in cancer patients with CIT has been deficient, resulting in it being consigned to off-label use [15, 16]; there have been no MPL agonists approved for CIT treatment, which suggests the need to develop effective therapeutics.

In this study, we developed and characterized the novel MPL agonist antibody, 2R13. This antibody is a fully human agonist antibody selected via a BaF3/MPL cell-based assay following phage display using human combinatorial antibody phage libraries. We found that 2R13 promoted megakaryopoiesis and platelet production both in vitro and in vivo. Furthermore, 2R13 significantly improved the platelet count in a 5-fluorouracil (5-FU)-induced thrombocytopenia mouse model, suggesting potential as a therapeutic antibody for CIT.

## Materials and methods

### Cell lines and cell culture

The Expi293F™ cell line (Gibco, Grand Island, NY, US) was maintained according to the manufacturer’s recommendations. The murine pre-B cell line, BaF3, was obtained from Dr. Arthur J. Sykowski (Beth Israel Deaconess Medical Center, Boston, MA, US) and cultured in RPMI-1640 medium (Lonza, Walkersville, MD, US) supplemented with 10% fetal bovine serum (FBS) and 5% WEHI‐3B cell-conditioned medium (WEHI-CM, as a source of IL-3). BaF3 cells were stably transfected with the pCMV-human MPL plasmid (Origene, Rockville, MD, US) to establish a BaF3/MPL cell line expressing human MPL. The surface expression of human MPL was confirmed using flow cytometry with anti-CD110-APC-conjugated antibody (MiltenyiBiotec, Auburn, CA, US). The acute megakaryoblastic leukemia cell line, M07e, was purchased from the German Collection of Microorganisms and Cell Cultures (DSMZ) and grown in IMDM medium supplemented with 10% FBS and 10 ng/mL recombinant human IL-3. Normal human platelets were obtained within 1–2 days after expiration dates from the Blood Bank at Kosin University Gospel Hospital (Busan, Republic of Korea).

### Antibody selection

The naïve human combinatorial antibody phage libraries (diversity ≈ 10^9^) were obtained from Scripps Research (La Jolla, CA, US). The overall antibody panning was done as described in a previous study [17]. Briefly, Dynabeads™ M-270 Epoxy beads (Invitrogen, San Diego, CA, US) were coupled with recombinant human MPL (rhMPL, R&D Systems, Minneapolis, MN, US) at 37 °C overnight with end-over-end rotation. The beads were blocked using MPBST (2% skim milk and 0.05% Tween 20 in phosphate-buffered saline [PBS]) at room temperature for 1 h. After adding the antibody phage libraries, the blocked beads-libraries mixture was incubated at room temperature with end-over-end rotation for 1 h. Subsequently, the supernatant was removed, and the beads were rinsed three times with PBST (0.05% Tween 20 in PBS). The bound phages were eluted using 0.2 M glycine–HCl (pH 2.2) and subsequently neutralized with 2 M Tris–HCl (pH 8.0).

### Enzyme-linked immunosorbent assay (ELISA)

ELISA plates were coated with rhMPL (R&D Systems) or bovine serum albumin (BSA; negative control; BD Biosciences, Franklin Lakes, NJ, US) at 4 °C overnight. Each well was washed with PBS and blocked with MPBS (5% skim milk in PBS) at 37 °C for 1 h. Next, the phages were added, and incubated at 37 °C for 2 h. After washing five times with PBS, the bound phages were detected using anti-M13 phage-HRP-conjugated antibody (1:2000; Sino Biological Inc, Chesterbrook, PA, US). Absorbance was measured at 450 nm using a VersaMax™ Microplate Reader (Molecular Devices, San Jose, CA, US). The 2R13 was detected using anti-human IgG Fc-HRP-conjugated antibody (1:2000; Abcam, Cambridge, MA, US).

### Antibody expression and purification

The sequences of complementarity-determining regions (CDRs) of selected clones were analyzed using the IMGT numbering tool web server, VBASE2. The CDR sequences of each clone were inserted into the pFuse-Fc expression vector (Invivogen, San Diego, CA, US). The antibodies were produced using the Expi293F™ cell expression system (Thermo Fisher Scientific, Waltham, MA, US). The soluble single-chain fragment variable-fragment crystallizable (scFv-Fc) antibodies in the supernatant were purified over the HiTrap protein G HP column (Cytiva, Marlborough, MA, US) using the ÄKTAprime Plus system (Cytiva).

### Cell proliferation assay

Each cell was incubated in the culture medium without WEHI-CM for 48 h. For activity-based antibody selection, BaF3/MPL (1 × 10^5^ cells/mL) cells were incubated with rhTPO (R&D Systems) or antibodies. Cell proliferation was measured by the CellTiter® 96 AQueous One Solution Cell Proliferation Assay System (Promega, Madison, WI, US) with absorbance at 490 nm. To validate 2R13 agonistic activity, BaF3 (1 × 10^5^ cells/mL), BaF3/MPL (1 × 10^5^ cells/mL), and M07e (5 × 10^5^ cells/mL) were incubated with rhTPO (PeproTech, Rocky Hill, NJ, US) or 2R13. BaF3 and BaF3/MPL cell proliferation was measured by Cell Titer-Glo® Luminescent Cell Viability assay kit (Promega) using Victor3 V 1420 Multilabel Counter (Perkin Elmer Inc, Waltham, MA, US). M07e cell proliferation was measured by Cell Counting Kit-8 (GlpBio, Montclair, CA, US) with absorbance at 460 nm.

### Surface plasmon resonance (SPR) spectrometry

The binding kinetics of 2R13 were measured using the iMSPR mini-instrument (icluebio, Seongnam, Republic of Korea). rhMPL (R&D Systems) or recombinant mouse MPL (rmMPL, R&D Systems) was immobilized on the research-grade carboxylic (COOH) sensor chip (icluebio) by using 10 mM sodium acetate buffer (pH 4.0) at approximately 500 response units (RUs). The chip was blocked with 1 M ethanolamine (pH 8.0) and two-fold serial dilutions (256–16 nM) of 2R13 were individually injected. Data were analyzed using the iMSPR analysis software (TraceDrawer; icluebio).

### Western blotting

BaF3/MPL cells (4 × 10^5^ cells/mL) and human platelets were serum-starved in the culture medium with 0.5% FBS overnight or for 3 h, respectively. The cells and platelets were stimulated with rhTPO or 2R13. The cell lysates were quantified using the bicinchoninic acid (BCA) protein assay kit (Thermo Fisher Scientific) and boiled with sodium dodecyl sulfate (SDS)-polyacrylamide gel electrophoresis (PAGE) sample buffer at 95 °C for 5 min. The samples (10 μg/well) were separated using SDS-PAGE and transferred onto a nitrocellulose membrane (Millipore). Membranes were cut to enable blotting for multiple antibodies. The expression of proteins was detected using primary antibodies against the Janus family of tyrosine kinases (Jak2), p-Jak2, signal transducers and activators of transcription (Stat5), p-Stat5 (Y925), Stat3, p-Stat3 (Y705), Akt, p-Akt (S473), Erk and p‐Erk1/2 (Thr202/Tyr204), which were purchased from Cell Signaling Technology (Danvers, MA, US). Antibody against β‐actin was purchased from Novus Biologicals (Minneapolis, MN, US). The blot was imaged using Amersham™ Imager 600 (GE Healthcare Life Sciences, Chicago, IL, US).

### Reporter gene expression assay

BaF3/MPL cells (2 × 10^6^ cells/mL) were transfected with pGL4.52[luc2P/Stat5RE/Hygro] vector (Promega) using the SG Cell Line 4D-Nucleofector kit (Lonza) following the manufacturer’s protocol. The transfected cells (2 × 10^4^ cells/well) were incubated with rhTPO, control antibody, or 2R13 in the culture medium without WEHI-CM for 6 h. Luciferase (reporter gene) expression was measured using the Bright-Glo™ Luciferase Assay System (Promega) by an Envision™ multimode plate reader (PerkinElmer, Waltham, MA, US).

### PB-CD34+ cell isolation

Granulocyte colony-stimulating factor-mobilized human apheresis samples were obtained from healthy donors within the framework of standard procedures for hematopoietic stem and progenitor cell (HSPC) apheresis at Kosin University Gospel Hospital after obtaining written informed consent from the donors. Peripheral blood mononuclear cells were isolated by density gradient centrifugation with Ficolle-Hypaque (Sigma, Louis, MO, US) and subjected to PB-CD34^+^ cell immunoselection using the magnetic-activated cell sorting (MACS) CD34 MicroBead UltraPure kit (Miltenyi Biotec) according to the manufacturer’s protocol. The purity of the isolated PB-CD34^+^ cells was routinely higher than 95%, as determined using CytoFLEX Flow Cytometry (Beckman, Brea, CA, US) with anti-human CD34-PE-conjugated antibody.

### Megakaryopoiesis analysis

PB-CD34^+^ cells (4 × 10^4^ cells/mL) were incubated with rhTPO or 2R13 in serum-free expansion medium (SFEM, Stem Cell Technologies, Vancouver, BC, Canada) supplemented with recombinant human stem cell factor (25 ng/mL; PeproTech), recombinant human IL-6 (10 ng/mL; PeproTech), recombinant human IL-9 (10 ng/mL, PeproTech), and LDL (25 μg/mL, Stem Cell Technologies for up to 14 days. Next, the cells were harvested at each time point (days 4, 7, 11, and 14) and labeled with anti-CD41a-FITC-conjugated antibody (Miltenyi Biotec), anti-CD42b-PE-conjugated antibody (Miltenyi Biotec), or 7-amino-actinomycin D (AAD; eBioscience, San Diego, CA, US) at 4 °C for 30 min. After washing with PBS, the cells were analyzed using flow cytometry.

### Polyploidy analysis of MKs

PB-CD34^+^ cells cultured for 13 days were labeled with anti-CD41a-FITC-conjugated antibody at 4℃ for 30 min and washed with cold PBS. The labeled cells were fixed with 1% paraformaldehyde (PFA) at room temperature for 15 min and permeabilized with 70% methanol at − 20℃ for 1 h. The cells were then treated with RNase (10 μg/mL; Roche, Basel, Switzerland), stained with propidium iodide (PI; 10 μg/mL; Sigma) at room temperature for 30 min, and analyzed using flow cytometry.

### In vivo experiments

All experiments in mice were conducted in accordance with the animal experimental guidelines approved by the Institutional Animal Care and Use Committee (IACUC) at Inje University College of Medicine (Busan, Republic of Korea). 8 to 10-week-old BALB/c female mice (Nara-Biotech, Seoul, Republic of Korea) were subcutaneously injected with 0.1% BSA-PBS or rhTPO for seven consecutive days, or once with 2R13. To establish the thrombocytopenia in vivo model, the mice were intraperitoneally injected with 5-FU (150 mg/kg; Sigma) 1 h before treatment. Each mouse was anesthetized with an intraperitoneal injection of ketamine (90 mg/kg) and xylazine (10 mg/kg); then, blood samples (50 μL) were collected from the retro-orbital sinus into ethylenediaminetetraacetic acid (EDTA) capillary tubes (Marienfeld, Germany). The blood was transferred into an EDTA tube prefilled with 2.5 mM EDTA buffer. Platelets and WBCs were counted by EONE Laboratories (Incheon, Republic of Korea). To investigate a change in LSK^+^ cell count induced by 2R13, BM-derived cells were collected from the femurs and tibias. The cells were flushed with PBS and red blood cells were lysed in lysing buffer at room temperature for 1 min, followed by flow cytometric analysis.

### Flow cytometry

To validate the binding activity of 2R13 against MPL, BaF3 and BaF3/MPL cells (1 × 10^6^ cells/mL) were fixed with 4% PFA at 4 °C for 10 min. After washing with PBS, the cells were incubated with 2R13 or control antibody at 4 °C for 1 h, followed by anti-human IgG Fc-FITC-conjugated antibody (Abcam) at 4 °C for 1 h. To examine the percentage of LSK^+^ cells, BM cells were resuspended in PBS and incubated with PerCP-Cy5.5-labeled lineage antibody cocktail, anti-Sca-1-FITC-conjugated antibody, and anti-c-kit-APC-conjugated antibody (BD Biosciences) at 4 °C for 30 min. The samples were analyzed using the FACSCanto™ II Flow Cytometry and FlowJo software.

### Statistical analysis

Data were analyzed using the GraphPad software (Prism v.9.0; San Diego, CA, US). All data are the mean ± standard deviation (SD). Between-group differences were analyzed using the unpaired t-test. Statistical significance between multiple groups was analyzed using one-way analysis of variance (ANOVA) or two-way ANOVA, followed by Dunnett’s multiple comparison test.

## Results

### Selection of the human anti-MPL agonist antibody

**Fig. 1:**
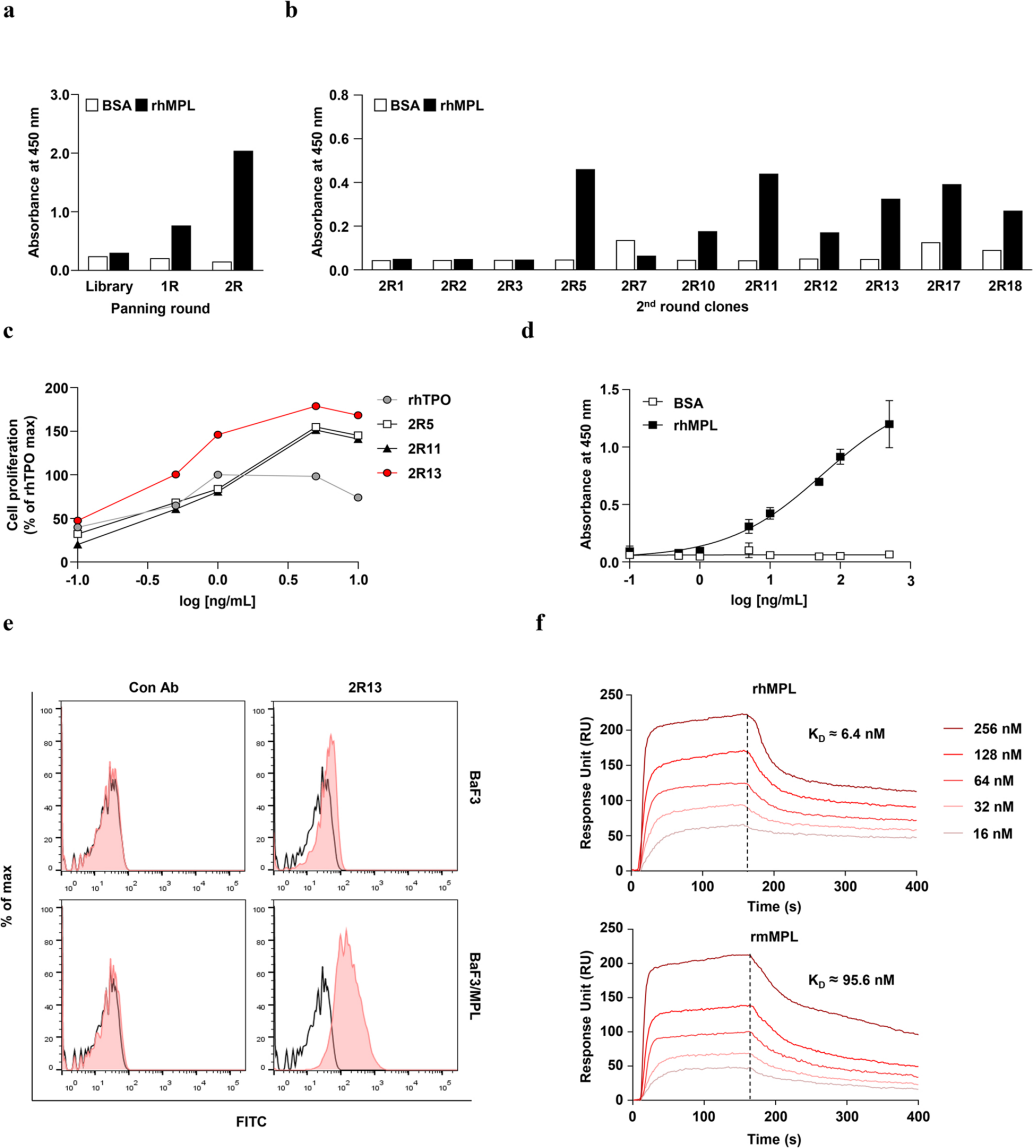
Selection of agonist antibodies against MPL. **a** Polyclonal phage ELISA for confirming the enrichment of rhMPL-binding phage antibodies of each round of solution-phase panning. **b** Monoclonal phage ELISA for selecting rhMPL-specific binding antibodies. **c** BaF3/MPL cell proliferation assay for determining agonistic antibodies. BaF3/MPL cells were incubated with rhTPO (positive control), 2R5, 2R11, or 2R13 over the indicated concentration range. **d** ELISA for detecting 2R13 binding activity against rhMPL. The EC_50_ was calculated by fitting the data to a four-parameter logistic model using GraphPad Prism 9. Data are the mean ± SD (*n* = 3). **e** Flow cytometric analysis for detecting 2R13 binding specificity in BaF3 and BaF3/MPL cells. **f** SPR analysis of 2R13. Two-fold serially diluted 2R13 was injected into the captured rhMPL or rmMPL. Kinetic data from a representative experiment were fitted to a 1:1 binding model. The equilibrium dissociation constant (K_D_) has been presented

Antibodies binding to MPL were selected by solution-phase panning from the naïve human combinatorial antibody phage libraries. We performed two rounds of panning on rhMPL as the antigen and confirmed the enrichment of binding phages (Fig. 1a). We randomly selected 12 clones from the output phage pool of the second round and individually tested their binding to rhMPL. Seven clones (2R5, 2R10, 2R11, 2R12, 2R13, 2R17, and 2R18) bound to rhMPL with an OD_450 nm_ value approximately three-fold higher than that of BSA (Fig. 1b). To identify potential agonist antibodies among these clones, we tested whether each clone in the scFv-Fc format induced the proliferation of TPO-responsive cells (BaF3/MPL). We found that three clones (2R5, 2R11, and 2R13) induced BaF3/MPL cell proliferation, with 2R13 exhibiting the most potent activity (Fig. 1c). Furthermore, we analyzed the binding activity of 2R13 to MPL. The 2R13 bound to rhMPL with an EC_50_ of 55.28 ng/mL (Fig. 1d) and specifically interacted with BaF3/MPL cells but not BaF3 cells (Fig. 1e). MPL is conserved between mice and humans with over 80% homology at the amino acid level [18]. As the results of analyzing the cross-reactivity, 2R13 interacted with both rmMPL (*K*_D_ ≈ 95.6 nM) and rhMPL (*K*_D_ ≈ 6.4 nM) (Fig. 1f).

### 2R13 promoted cell proliferation by activating the MPL signaling pathways in mouse and human cells

**Fig. 2:**
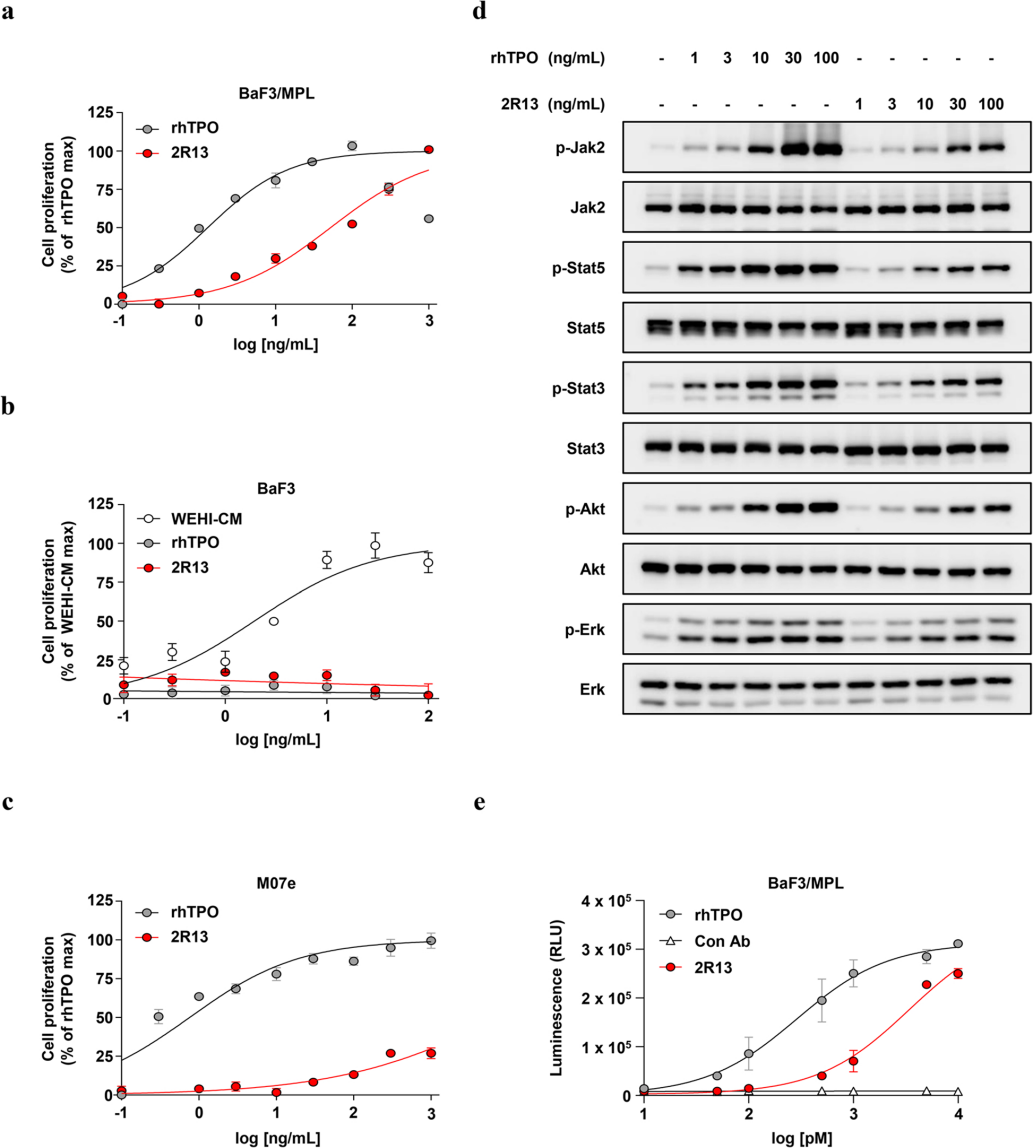
Agonistic activity and MPL signal transduction in mouse and human cells by 2R13. **a**-**c** Cell proliferation assay for detecting the agonistic activity of 2R13. **a** BaF3/MPL cells were incubated with rhTPO (positive control) or 2R13, and **b** Parental BaF3 cells were incubated with WEHI-CM (positive control), rhTPO, or 2R13, over the indicated concentration range (*n* = 3). The EC_50_ was calculated by fitting the data to a four-parameter logistic model using GraphPad Prism 9. **c** M07e cells were incubated with rhTPO (positive control) or 2R13 over the indicated concentration range (*n* = 3). **d** Western blotting of BaF3/MPL cells stimulated with rhTPO or 2R13 over the indicated concentration range for 30 min, using the indicated antibodies. **e** Luciferase reporter assay in BaF3/MPL cells transfected with pGL4.52 [luc2P/Stat5RE/Hygro] plasmids. Cells were stimulated with rhTPO (positive control), control antibody (negative control), or 2R13 over the indicated concentration range for 6 h (*n* = 2). Data are the mean ± SD. WEHI-CM, WEHI-3B cell-conditioned medium

To further evaluate the agonistic activity of 2R13, we examined 2R13-dependent cell proliferation in three cell types. The 2R13 showed lower potency than rhTPO with an EC_50_ of 1.3 ng/mL but promoted BaF3/MPL cell proliferation with an EC_50_ of 52.44 ng/mL (Fig. 2a). In contrast, the proliferation of wild-type BaF3 cells was not affected by rhTPO or 2R13; thus, MPL-specific proliferation was noted in BaF3/MPL cells (Fig. 2b). The 2R13 also induced cell proliferation in the M07e cell line, a human leukemic cell line that endogenously expresses MPL and requires TPO as a survival and proliferation factor (Fig. 2c) [19]. To determine the mechanism underlying the promotion of receptor-mediated cell proliferation by 2R13, we examined MPL-dependent signaling in BaF3/MPL cells. TPO homodimerizes MPL and activates Stat, Akt, and Erk via the receptor-associated tyrosine kinase, Jak2 [20]. The 2R13 showed patterns similar to those for rhTPO-induced protein phosphorylation in a dose-dependent manner (Fig. 2d and uncropped blots in Supplementary Fig. 7). We further validated 2R13 signal transduction via luciferase expression mediated by Stat5 activation (Fig. 2e).

### 2R13 stimulated MK differentiation from PB-CD34+ cells

**Fig. 3:**
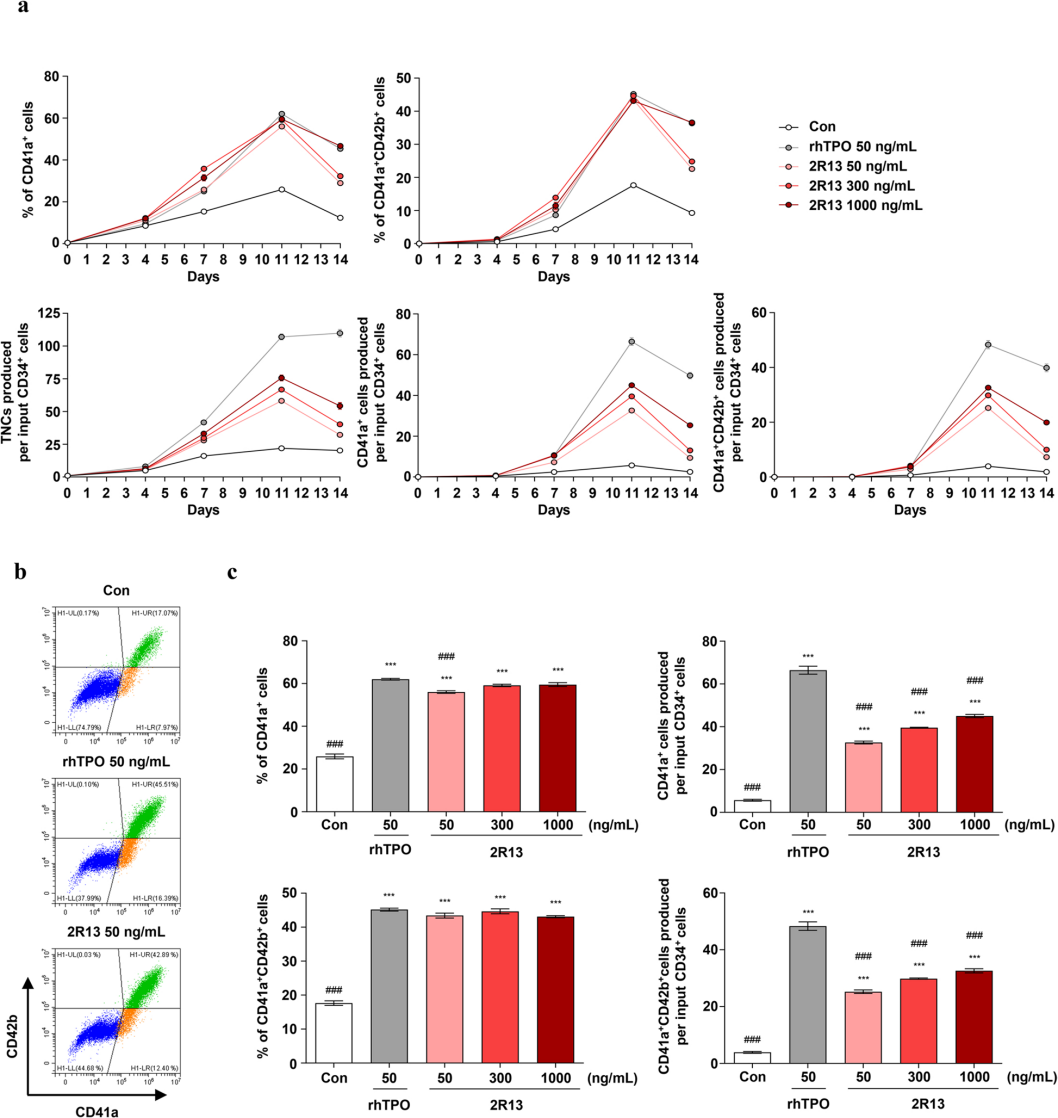
Effect of 2R13 on MK differentiation in PB-CD34^+^ cells. PB-CD34^+^ cells were stimulated with rhTPO or 2R13 at the indicated concentrations for 14 days. On days 4, 7, 11, and 14, the number of total nucleated cells was counted using an ADAM-MC automated mammalian cell counter, and the cells were analyzed using flow cytometry with anti-CD41a-FITC-conjugated and anti-CD42b-PE-conjugated antibodies. **a** Percentage and number of total nucleated, CD41a^+^, and CD41a^+^CD42b^+^ cells. The number of cells produced per input of PB-CD34^+^ cells was calculated by multiplying the number of total nucleated cells with the percentage of CD41a^+^ or CD41a^+^CD42b^+^ cells. **b** Representative flow cytometric analysis of PB-CD34^+^ cell differentiation on day 11. **c** Percentage (left panels) and number (right panels) of CD41a^+^ and CD41a^+^CD42b^+^ cells on day 11. Data are the mean ± SD (*n* = 3). One-way ANOVA was used for statistical analysis. ***p < 0.001 vs control; ###*p* < 0.001 vs rhTPO; TNC, total nucleated cells

To measure the effect of 2R13 on MK differentiation from peripheral blood (PB)-derived CD34^+^ cells, we cultured PB-CD34^+^ cells for 14 days in a megakaryocytic differentiation medium supplemented with either rhTPO or 2R13. We investigated two populations, immature MKs (CD41a^+^) and mature MKs (CD41a^+^CD42b^+^), from the differentiated cells on days 4, 7, 11, and 14. On day 11, the ratio of both MK populations and the number of total nucleated cells reached a peak by rhTPO or 2R13 (Fig. 3a); 2R13 led to the production of CD41a^+^ and CD41a^+^CD42b^+^ cells in a proportion similar to that obtained with rhTPO (Fig. 3b and left panels of 3c). Analysis of the number of cells produced per input of PB-CD34^+^ cells showed that the cell counts induced by 2R13 were lower than those induced by rhTPO; The number of CD41a^+^ cells increased 5.7-fold at 50 ng/mL, 6.9-fold at 300 ng/mL and 7.9-fold at 1,000 ng/mL 2R13 vs 11.7-fold at 50 ng/mL rhTPO, compared with the control. The number of CD41a^+^CD42b^+^ cells increased 6.5-fold at 50 ng/mL, 7.7-fold at 300 ng/mL, and 8.4-fold at 1,000 ng/mL vs 12.5-fold at 50 ng/mL rhTPO, compared with the control (Fig. 3c, right panels). We obtained similar results with PB-CD34^+^ cells derived from additional donors (donor 2 and 3; Supplementary Fig. 1 and Supplementary Fig. 2), demonstrating the functional effectiveness of 2R13.

### 2R13 promoted MK maturation during MK differentiation from PB-CD34+ cells

**Fig. 4:**
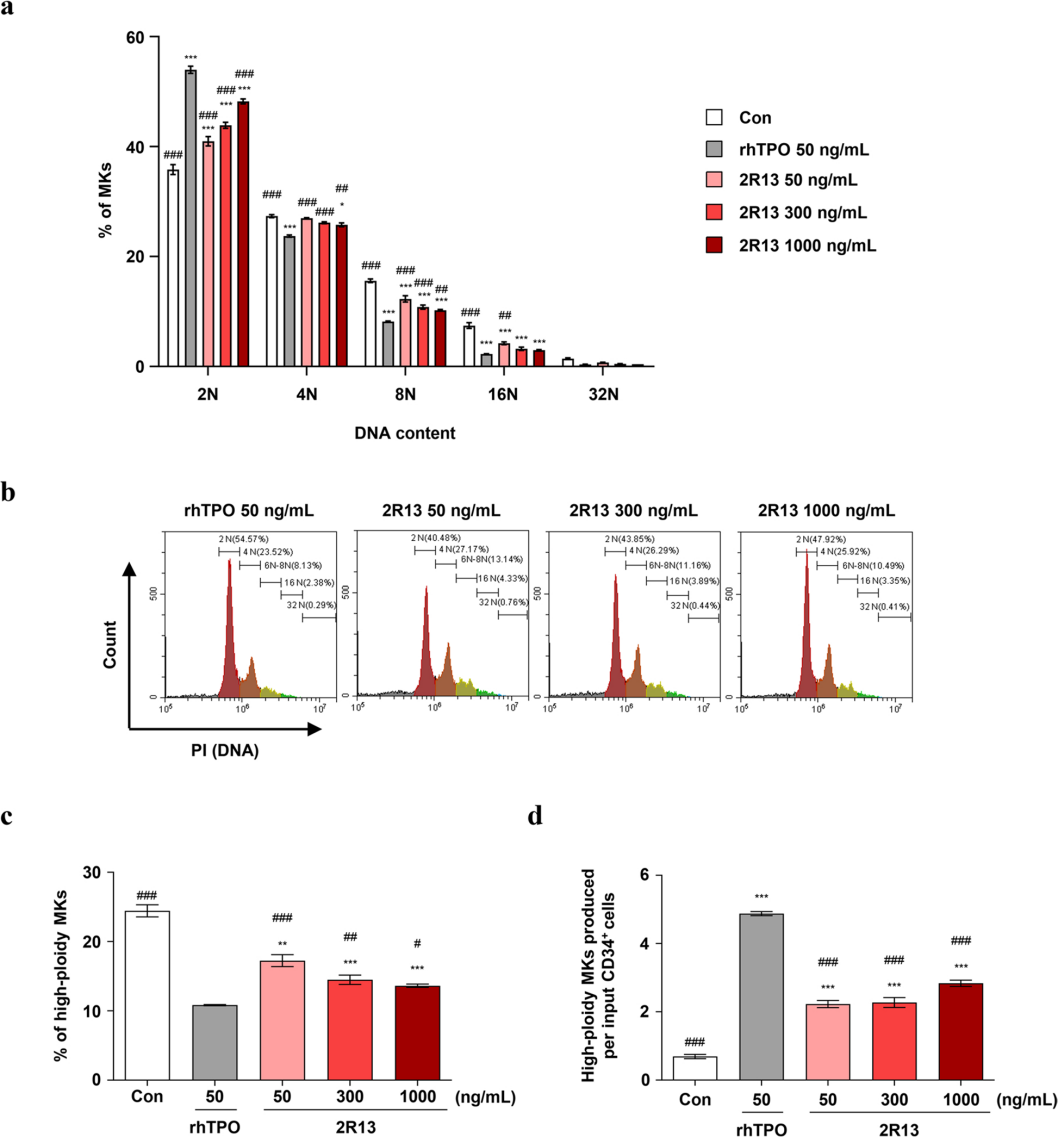
Effect of 2R13 on MK polyploidization in PB-CD34^+^ cells. PB-CD34^+^ cells were stimulated with rhTPO or 2R13 at the indicated concentrations for 13 days. Differentiated cells were analyzed using flow cytometry with anti-CD41a-FITC-conjugated antibody, followed by propidium iodide staining. **a** Ploidy status of CD41a^+^ cells. **b** Representative flow cytometric profile of ploidy levels. **c**, **d** Percentage and number of high-ploidy (≥ 8N) MKs. The number of cells produced per input of PB-CD34^+^ cells was calculated by multiplying the number of total nucleated cells with the percentage of high-ploidy CD41a^+^ cells. Data are the mean ± SD (*n* = 3). One-way and two-way ANOVA were used for statistical analysis. *p < 0.05, **p < 0.01, and ***p < 0.001 vs control; #*p* < 0.05, ##*p* < 0.01, and ###*p* < 0.001 vs rhTPO

During MK differentiation, committed MK progenitors initiate proliferation through a mitotic process; however, during the late phases of differentiation, the cells switch to an endomitotic process to achieve polyploid and undergo maturation [21]. Therefore, we investigated the polyploidy level achieved by 2R13 to examine the mechanism underlying the contribution of this antibody to MK differentiation. PB-CD34^+^ cells were cultured for 13 days in the megakaryocytic differentiation medium supplemented with either rhTPO or 2R13. The 2R13 led to a lesser proportion of 2N MKs and greater proportions of 4N and ≥ 8N MKs than those obtained with rhTPO (Fig. 4a, b). The ratio of high-ploidy MKs (≥ 8N) was 17.2% at 50 ng/mL, 14.5% at 300 ng/mL, and 13.6% at 1,000 ng/mL of 2R13 vs 10.8% at 50 ng/mL of rhTPO (Fig. 4c). We observed similar results for PB-CD34^+^ cells derived from donor 2 (Supplementary Fig. 3a-c), whereas cells derived from donor 3 showed a slightly different pattern of MK ploidy achieved by 2R13; the ratio of each ploidy MK was similar to that of rhTPO (Supplementary Fig. 4a-c). Furthermore, we examined the number of cells produced per input of PB-CD34^+^ cells. The number of 2R13-induced high-ploidy MKs was lower than that of rhTPO-induced high-ploidy MKs, with 2.2 at 50 ng/mL, 2.3 at 300 ng/mL, and 2.8 at 1,000 ng/mL of 2R13 vs 4.9 at 50 ng/mL of rhTPO (Fig. 4d). Similar results were obtained with PB-CD34^+^ cells derived from additional donors (Supplementary Fig. 3d and Fig. 4d). Taken together, 2R13 did not increase the number of total nucleated cells as much as rhTPO, but rather induced a greater high-ploidy MK proportion than rhTPO. Based on these findings, we conclude that 2R13 mainly supports MK maturation during MK differentiation.

### 2R13 stimulated the MPL signaling pathways in human platelets

**Fig. 5:**
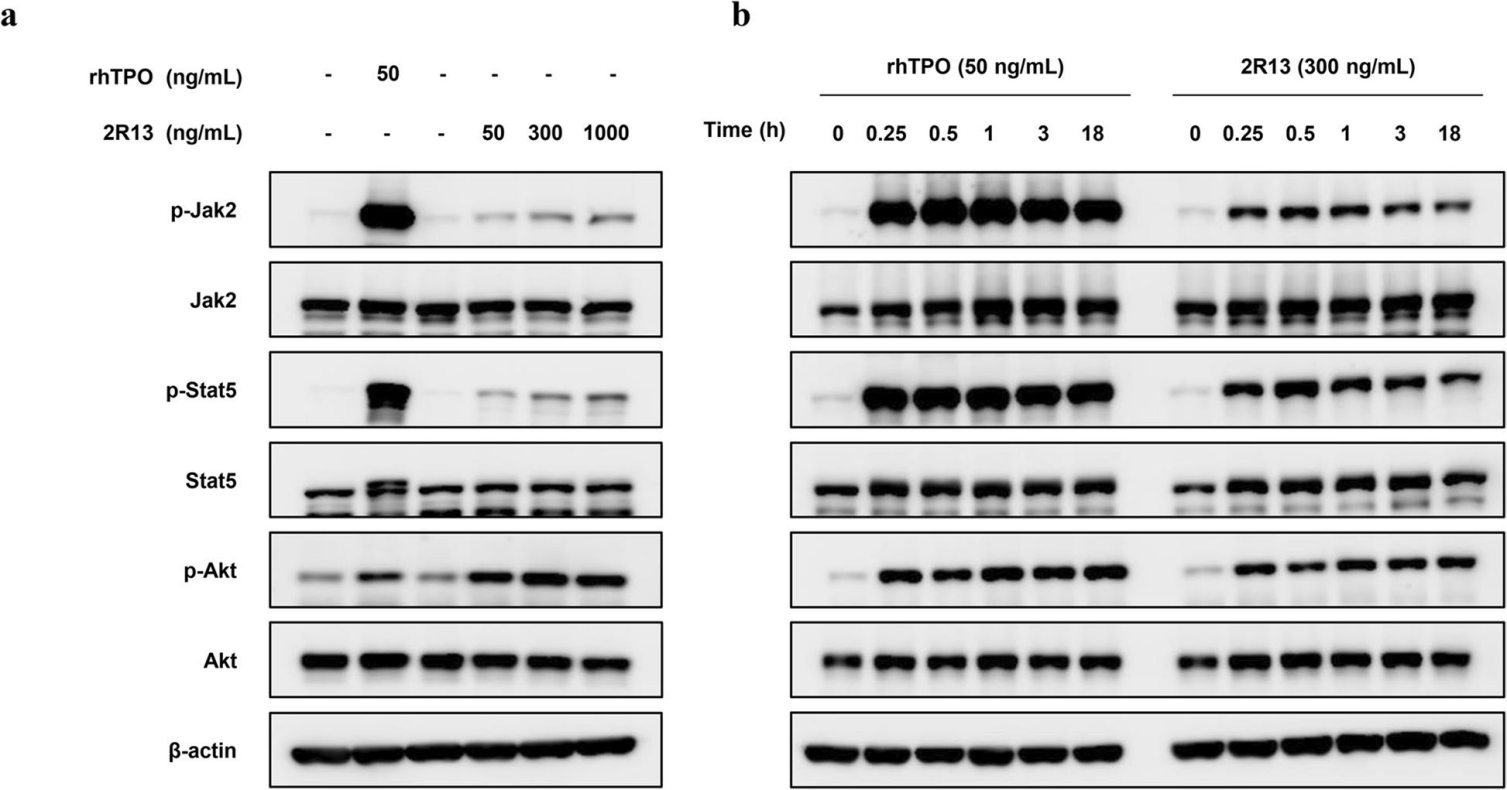
MPL signal transduction by 2R13 in human platelets. Western blotting of human platelets stimulated with rhTPO or 2R13. **a** rhTPO or 2R13 was added at the indicated concentrations for 30 min. **b** rhTPO (50 ng/mL) or 2R13 (300 ng/mL) was added over the indicated period. Total lysates were analyzed by immunoblotting with the indicated antibodies and β-actin was used as a loading control

The 2R13 stimulated the proliferation and the intracellular signaling pathways in BaF3/MPL cells and M07e cells, which express ectopic and endogenous MPL, respectively (see Fig. 2). We then evaluated whether 2R13 activated the MPL-dependent signaling in primary human platelets. The 2R13 dose-dependently stimulated phosphorylation of Jak2, Stat5, and Akt, similar to rhTPO (Fig. 5a and uncropped blots in Supplementary Fig. 8). In addition, rhTPO or 2R13 led to persistently high phosphorylation levels for up to 18 h (Fig. 5b and uncropped blots in Supplementary Fig. 8). We confirmed a similar pattern of signal activation by 2R13 in platelets derived from addition donors (donor 2 and 3; Supplementary Fig. 5 and uncropped blots in Supplementary Fig. 9 and Fig. 10).

### 2R13 promoted platelet productionin vivo

**Fig. 6:**
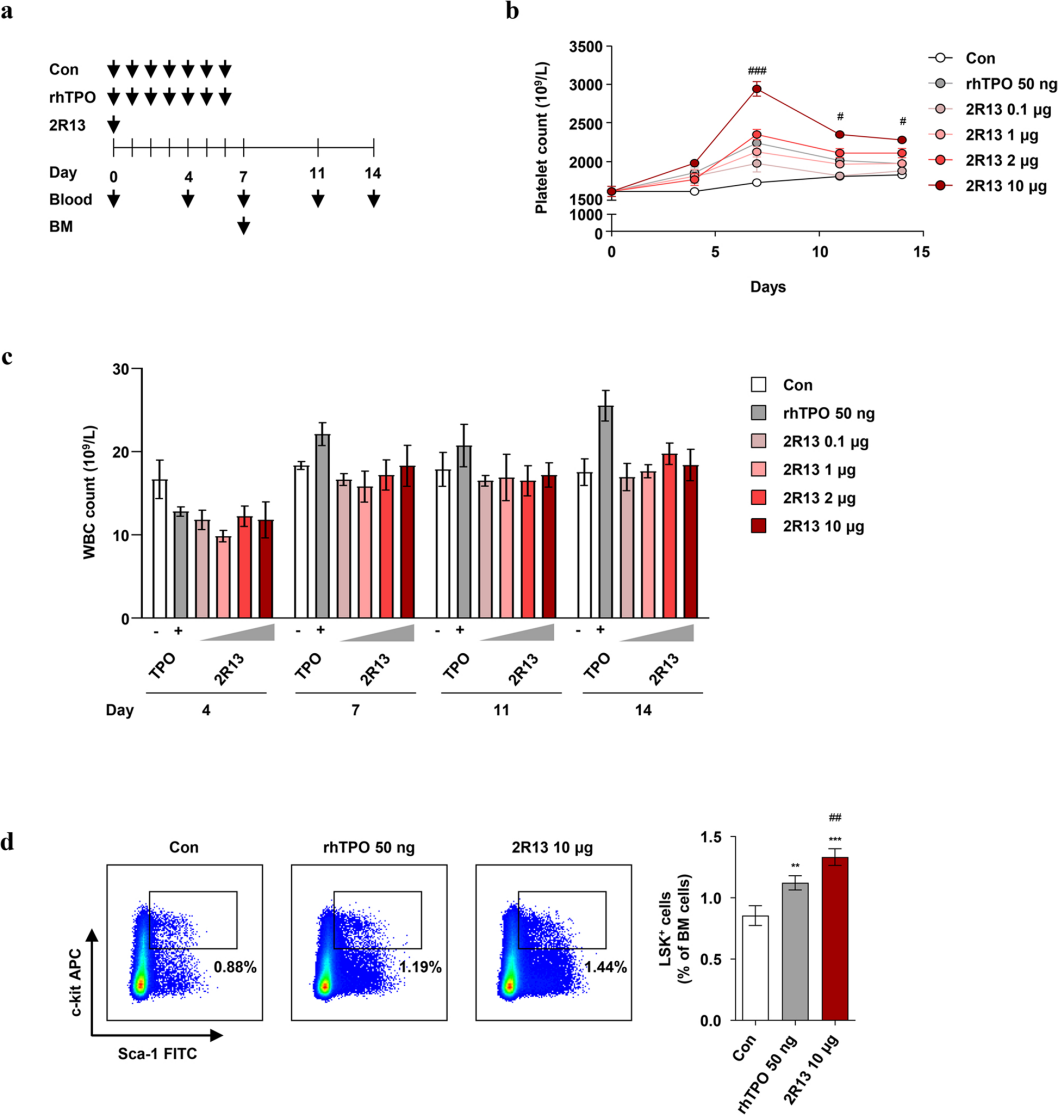
Platelet production induced by 2R13 in mice. **a** Schematic of the experimental design. BALB/c mice were subcutaneously injected with 0.1% BSA-PBS (control) or rhTPO (50 ng) for seven consecutive days, or once with 2R13 (0.1, 1, 2, or 10 μg). Blood was collected on the indicated days over 14 days. To analyze BM-derived LSK^+^ cells, BALB/c mice were subcutaneously injected with 0.1% BSA-PBS (control) or rhTPO (50 ng) for seven consecutive days, or once with 2R13 (10 μg). On day 7 post-initiation of treatment, BM cells were obtained after sacrificing the mice and were incubated with LSK^+^ cell markers (PerCP-Cy5.5-labeled lineage antibody cocktail, anti-Sca-1-FITC antibody, and anti-c-kit-APC antibody), following which they were analyzed using flow cytometry. Quantification of **b** platelet and **c** WBC counts. Data are the absolute count mean ± SD (*n* = 4/group). **d** Flow cytometric analysis of BM-derived LSK^+^ cells. Data are the mean ± SD (*n* = 4/group). The unpaired t-test was used for statistical analysis. **p < 0.01 and ***p < 0.001 vs control; #*p* < 0.05, ##*p* < 0.01, and ###*p* < 0.001 vs rhTPO

To determine whether 2R13 promoted platelet production due to MK differentiation, we monitored the platelet counts in BALB/c mice for 14 days. The mice were injected with rhTPO for seven consecutive days, or once with 2R13, and blood was collected twice a week post-initiation of treatment (Fig. 6a). The platelet counts in 2R13-injected mice gradually increased in a dose-dependent manner from day 4, reached the highest level on day 7, and then decreased. The 10 µg of 2R13 remarkably increased the platelet level on day 7, maintaining a higher count until day 14 compared with that obtained by rhTPO (Fig. 6b). These data demonstrate that 2R13 promotes platelet production following MK differentiation induction, which confirms the relevance of our in vitro observations. In contrast, the 2R13-induced WBC production did not show significant changes during this period (Fig. 6c). TPO is also involved in the regulation of hematopoietic stem cells (HSCs) and the proliferation of primitive hematopoietic progenitor cells [22]. Therefore, we examined the effect of 2R13 on HSPCs in mice. On day 7 post-initiation of treatment, we found an increased percentage of LSK^+^ cells (Lin^−^/Sca-1^+^/c-kit^+^) in BM; therefore, 2R13 did cause LSK^+^ cells proliferation (Fig. 6d and Supplementary Fig. 6).

### 2R13 ameliorated 5-FU-induced thrombocytopenia

**Fig. 7:**
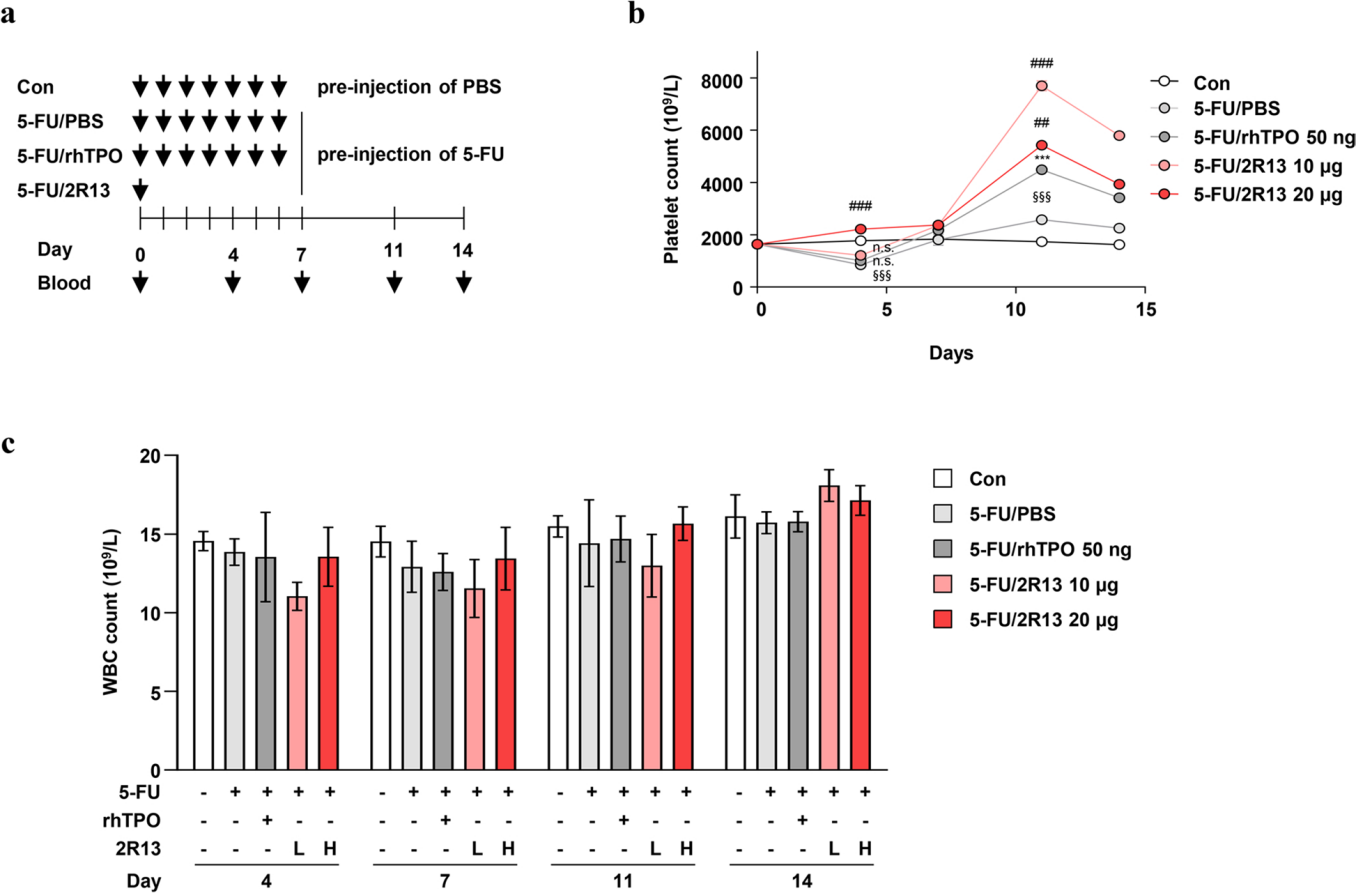
Platelet production induced by 2R13 in mice with 5-FU-induced thrombocytopenia. **a** Schematic of the experimental design. BALB/c mice were intraperitoneally injected with 0.1% BSA-PBS (control) or 5-FU (150 mg/kg). After 1 h, mice in the control and 5-FU/PBS groups were subcutaneously injected with 0.1% BSA-PBS for seven consecutive days over 14 days. Mice in the 5-FU/rhTPO and 5-FU/2R13 groups were subcutaneously injected with rhTPO for seven consecutive days or once with 2R13, respectively, over 14 days. Quantification of **b** platelet and **c** WBC counts. Data are the absolute count mean ± SD (*n* = 4/group). One-way ANOVA was used for statistical analysis. ***p < 0.001 vs 5-FU/PBS; ##*p* < 0.01 and ###*p* < 0.001 vs 5-FU/rhTPO; §§§*p* < 0.001 vs control; n.s., not significant; L, low concentration; H, high concentration

We evaluated the therapeutic potential of 2R13 against CIT. The mice were injected with 5-FU 1 h before treatment of either rhTPO or 2R13 (Fig. 7a). The platelet counts were significantly reduced on day 4 (by 5-FU) and returned to control levels from day 7 onwards. The 2R13-induced platelet production peaked on day 11. The produced platelets maintained a higher count than that obtained with rhTPO until day 14. In particular, 20 µg of 2R13 rescued the platelet count to a level comparable to that in the control mice from day 4, but it did not produce more platelets than 10 µg of 2R13 from day 7 onwards (Fig. 7b). The 5-FU and 2R13 did not significantly change the WBC count during this period (Fig. 7c). Collectively, our results show that a single injection of 2R13 may help relieve CIT by effectively promoting platelet production.

## Discussion

MPL agonists have recently been developed and approved by the FDA for the treatment of immune thrombocytopenia (ITP), aplastic anemia, hepatitis C-associated thrombocytopenia, and perioperative thrombocytopenia. However, none have been approved for CIT treatment [23–25]. Owing to CIT, treatment delay and decrease in administered doses result in reduced RDI, which lowers chemotherapy efficacy and affects patient survival outcomes. Thus, rapid and constant recovery of the platelet count during chemotherapy cycles without impacting the ongoing cancer therapy or RDI is crucial for CIT management. Our current findings suggest that 2R13, a human agonist antibody against MPL, is a potential therapeutic agent for CIT.

Among the three known MPL-associated signaling pathways, the Akt and Erk1/2 pathways play determinant roles in MK maturation and platelet formation. Akt signaling appears to be activated throughout MK differentiation. In contrast, Erk signaling appears to be strongly activated during the early stage and turned off during the late stages of MK differentiation [26, 27]. Previous research showed that romiplostim induces greater Akt signaling than that obtained with endogenous TPO, stimulating MK proliferation without parallel stimulation of MK maturation [28]. These findings indicate that regulation of cellular signaling by endogenous TPO and MPL agonists involves different mechanisms. The 2R13 phosphorylated Stat3/5, Akt, and Erk in similar to those of rhTPO in BaF3/MPL cells, but differing patterns were observed in promoting MK differentiation from PB-CD34^+^ cells. rhTPO increased the proportion of high-ploidy MKs by simultaneously stimulating MK proliferation (increase in 2N MK ratio) and differentiation. In contrast, 2R13 induced less MK proliferation but a greater high-ploidy MK ratio than that of rhTPO in PB-CD34^+^ cells derived from two out of three donors. These results indicate that 2R13 mainly supports polyploidization and maturation rather than proliferation during MK differentiation. However, to elucidate the precise mechanism underlying the involvement of 2R13 in MK differentiation and platelet production, further studies are required for more donor-derived PB-CD34^+^ cells.

A monoclonal antibody is one of the predominantly used therapeutic modalities because of its preferred biophysical properties, including high specificity, stability, long half-life, and low immunogenicity. The long half-life ensures durable effects with less frequent administration. We found that a single injection of 2R13 potently induced platelet production in the mouse model. In particular, high-dose 2R13 (20 μg) helped ameliorate the 5-FU-induced thrombocytopenia from day 4. Treatment with 2R13 sustained a higher platelet count than that obtained using rhTPO injection for seven consecutive days during the observation period, thereby effectively relieving thrombocytopenia. These data imply that 2R13 is a potential therapeutic agent that would enable CIT management, which requires rapid and continuous improvement in platelet count during chemotherapy cycles.

TPO also plays a critical role in HSC maintenance and hematopoietic recovery. It synergizes with other early cytokines to promote HSPC survival and proliferation, supporting their expansion and differentiation into multilineage immature progenitors/precursors in the BM [29]. We found that 2R13 increased the LSK^+^ cell count, but the WBC count did not change significantly during the study period. Based on these results, 2R13 is believed to promote HSPC proliferation and strongly drive thrombopoiesis within the BM, thereby mimicking the biological role of endogenous TPO. Chemotherapy may inhibit the development of other hematopoietic cells by impairing HSC capacity for self-renewal and differentiation, leading to BM failure. The damaged BM microenvironment of patients spontaneously recovers via HSPC regeneration after chemotherapy, while some patients receiving high-dose chemotherapy can only be restored via stem cell transplantation [30–33]. These functions of 2R13 mentioned above would aid in BM recovery and hematopoietic reconstitution, which suggests extended applicability in stem cell transplantation therapeutics.

This study is subject to several limitations. Firstly, 2R13 is a scFv-Fc format that is useful for characterizing potential scFvs chosen from phage display libraries along with Fc-mediated effector functions. Although some scFv-Fcs can be employed for therapy and diagnostics, the full-length IgG is still deemed the most appropriate format for clinical applications [34–36]. Therefore, in order to advance the clinical application of 2R13, its conversion to the full-length IgG format and optimization, including improving affinity, is required. Secondly, this study focused solely on the short-term impact of 2R13 over a 14-day period in a mouse model with thrombocytopenia. Prior research has shown that approximately 40% of solid tumor patients who received chemotherapy experience CIT recurrence, and the incidence of CIT positively correlates with the number of chemotherapy cycles received. When comparing the recurrence rate of CIT based on the frequency of romiplostim administration, intracycle dosing (administration of romiplostim only on chemotherapy off-weeks) resulted in a higher recurrence rate compared to weekly dosing. Romiplostim is generally administered on a weekly basis until the completion of chemotherapy treatment, highlighting the importance of achieving a sustained response for CIT management to maintain chemotherapy efficacy [37–39]. Future studies should evaluate the kinetics of platelet count with 2R13 treatment and its long-term consequences with regard to the continuation or discontinuation of treatment, utilizing detailed pharmacokinetic /pharmacodynamic profiling. Lastly, there is insufficient research on the direct effect of 2R13 on platelets. We observed that 2R13 activates MPL expressed in primary platelets via phosphorylation of the Jak2-Stat5 and Akt pathways. The TPO/MPL axis makes platelets susceptible to thrombus-forming and platelet-activating molecules, which contributes to the risk of venous thrombosis associated with malignancy [40–42]. Further studies are needed on platelet aggregation due to overactivation and changes in platelet surface markers because the impact of 2R13 on platelets is directly related to its safety as a therapeutic option for CIT.

## Conclusions

In summary, we developed a fully human agonist antibody, 2R13, that specifically binds to and activates MPL. Our data show that 2R13 promotes polyploidization and maturation during MK differentiation, followed by effective platelet production. In particular, the amelioration of 5-FU-induced thrombocytopenia with rapid recovery and continuous maintenance of platelet count by 2R13 demonstrates its potential as a promising therapeutic agent for CIT management.

## Supplementary Information

**Additional file 1:** **Supplementary Fig. 1.** Effect of 2R13 on MK differentiation in PB-CD34^+^cells isolated from donor 2.

**Additional file 2:** **Supplementary Fig. 2.** Effect of 2R13 on MK differentiation in PB-CD34^+^cells isolated from donor 3.

**Additional file 3:** **Supplementary Fig. 3.** Effect of 2R13 on MK polyploidization inPB-CD34^+^ cells isolated from donor 2.

**Additional file 4:** **Supplementary Fig. 4.** Effect of 2R13 on MK polyploidization inPB-CD34^+^ cells isolated from donor 3.

**Additional file 5:** **Supplementary Fig. 5.** MPL signal transduction induced by 2R13 inhuman platelets from donor 2 and donor 3.

**Additional file 6:** **Supplementary Fig. 6.** Flow cytometric analysis of LSK^+^ cells.

**Additional file 7:** **Supplementary Fig. 7.** Uncropped full-length images of westernblotting membranes.

**Additional file 8:** **Supplementary Fig. 8.** Uncropped full-length images of westernblotting membranes.

**Additional file 9:** **Supplementary Fig. 9.** Uncropped full-length images of westernblotting membranes.

**Additional file 10:** **Supplementary Fig. 10.** Uncropped full-length images of westernblotting membranes.

## Data Availability

All data generated or analyzed during this study are included in this published article and its supplementary information files.

## Abbreviations

CIT: Chemotherapy-induced thrombocytopenia
RDI: Relative dose intensity
TPO: Thrombopoietin
MPL: Myeloid proliferative leukemia
BM: Bone marrow
HSC: Hematopoietic stem cell
HSPC: Hematopoietic stem and progenitor cell
MK: Megakaryocyte
WBC: White blood cell
PB: Peripheral blood
5-FU: 5-Fluorouracil
WEHI-CM: WEHI-3B cell-conditioned medium
PBS: Phosphate-buffered saline
FBS: Fetal bovine serum
BSA: Bovine serum albumin
ANOVA: Analysis of variance
