# Supplementary material for: Thrombopoietin receptor agonist antibody for treating chemotherapy-induced thrombocytopenia
Source: BMC Cancer. 2023 May 31;23:490. doi: 10.1186/s12885-023-10975-3 (PMC10230746; doi:10.1186/s12885-023-10975-3)
Supplement: Supplementary file 1 — Additional file 1: Supplementary Fig. 1. Effect of 2R13 on MK differentiation in PB-CD34+cells isolated from donor 2. [file 12885_2023_10975_MOESM1_ESM.pdf]

Supplementary Fig. 1 Effect of 2R13 on MK differentiation in PB-CD34$^+$ cells isolated from donor 2

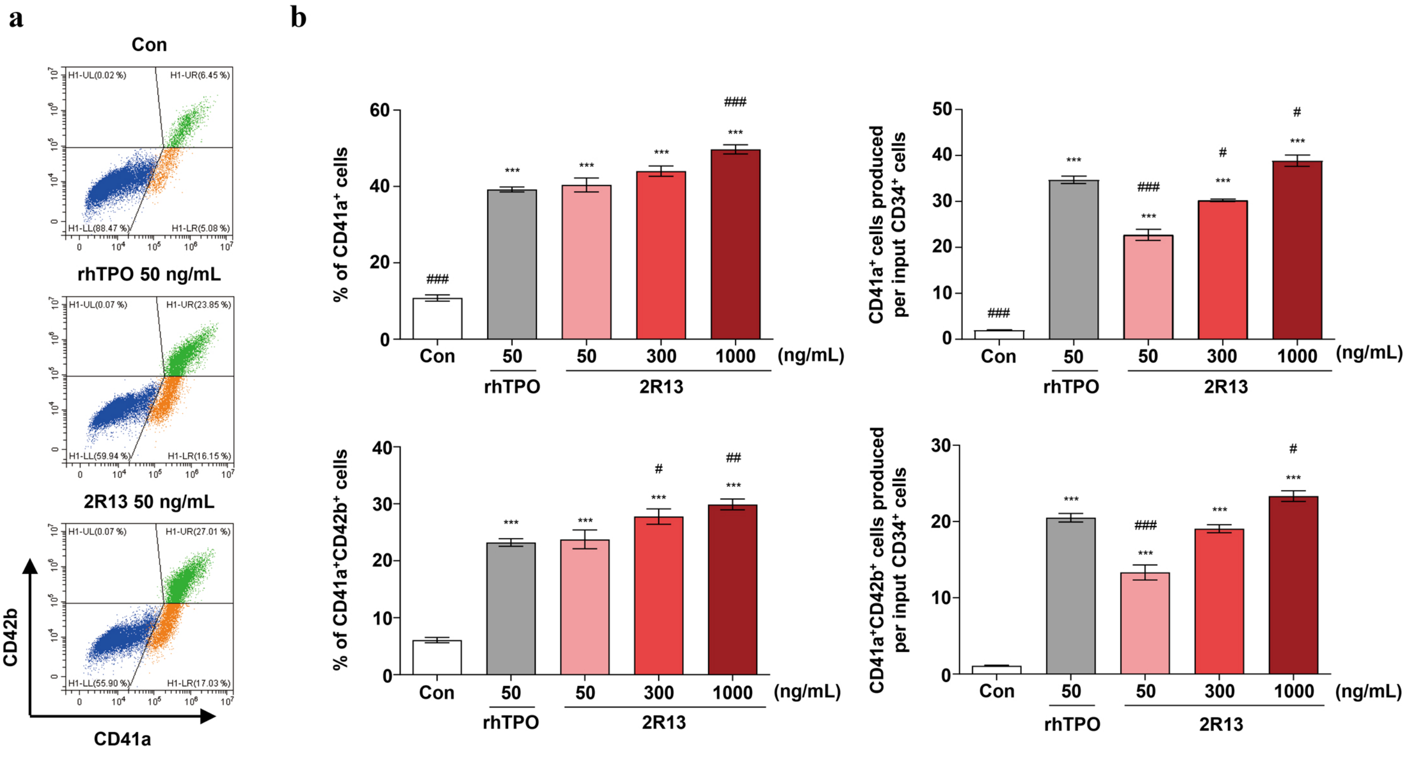

PB-CD34$^+$ cells derived from donor 2 were stimulated with rhTPO or 2R13 at the indicated concentrations for 11 days. **a** Representative flow cytometric analysis of PB-CD34$^+$ cell differentiation. **b** Percentage and number of CD41a$^+$ and CD41a$^+$CD42b$^+$ cells. The number of cells produced per input of PB-CD34$^+$ cells was calculated by multiplying the number of total nucleated cells with the percentage of CD41a$^+$ or CD41a$^+$CD42b$^+$ cells. Data are the mean ± SD (n = 3). One-way ANOVA was used for statistical analysis. ***$p < 0.001$ vs control; #$p < 0.05$, ##$p < 0.01$, and ###$p < 0.001$ vs rhTPO.
