# Supplementary material for: Thrombopoietin receptor agonist antibody for treating chemotherapy-induced thrombocytopenia
Source: BMC Cancer. 2023 May 31;23:490. doi: 10.1186/s12885-023-10975-3 (PMC10230746; doi:10.1186/s12885-023-10975-3)
Supplement: Supplementary file 2 — Additional file 2: Supplementary Fig. 2. Effect of 2R13 on MK differentiation in PB-CD34+cells isolated from donor 3. [file 12885_2023_10975_MOESM2_ESM.pdf]

Supplementary Fig. 2 Effect of 2R13 on MK differentiation in PB-CD34+ cells isolated from donor 3

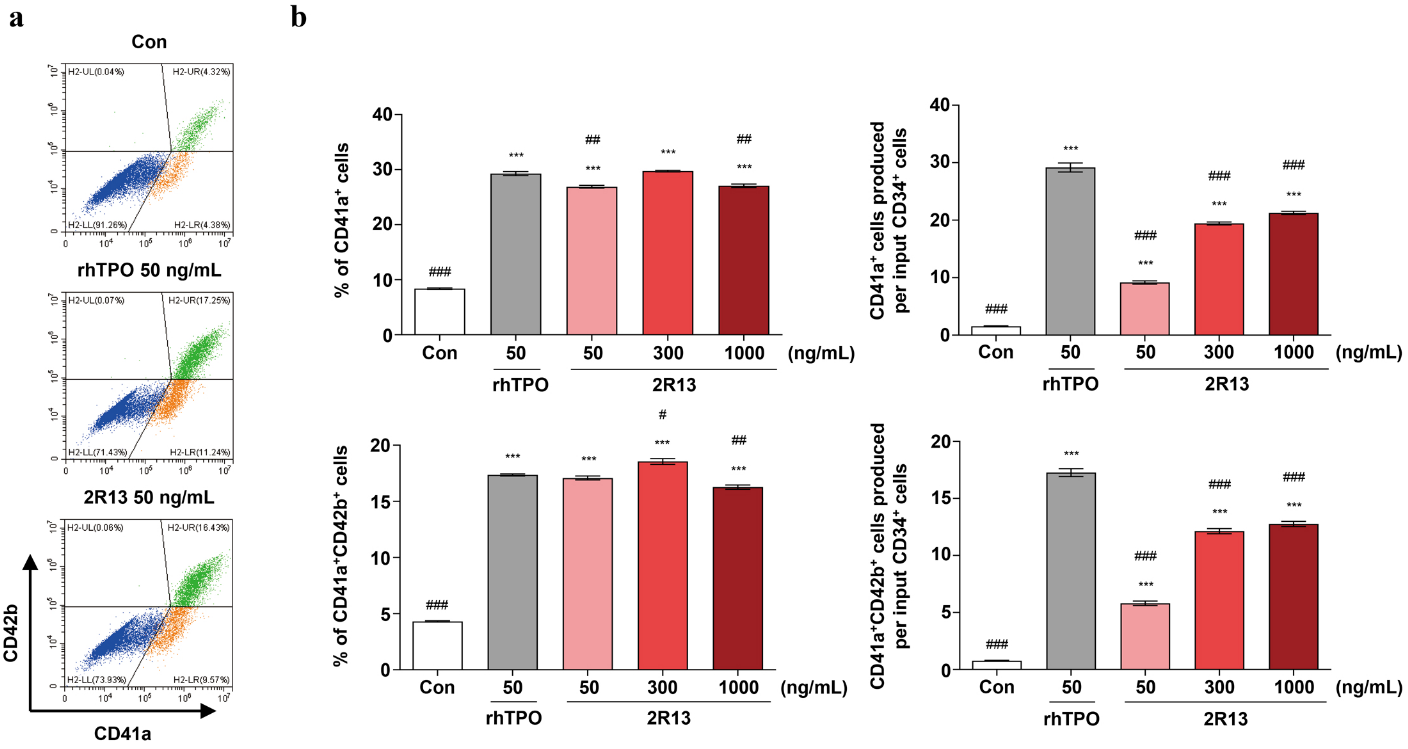

PB-CD34+ cells derived from donor 3 were stimulated with rhTPO or 2R13 at the indicated concentrations for 11 days. **a** Representative flow cytometric analysis of PB-CD34+ cell differentiation. **b** Percentage and number of CD41a+ and CD41a+CD42b+ cells. The number of cells produced per input of PB-CD34+ cells was calculated by multiplying the number of total nucleated cells with the percentage of CD41a+ or CD41a+CD42b+ cells. Data are the mean ± SD ($n = 3$). One-way ANOVA was used for statistical analysis. ***$p < 0.001$ vs control; #$p < 0.05$, ##$p < 0.01$, and ###$p < 0.001$ vs rhTPO.
