# Supplementary material for: Thrombopoietin receptor agonist antibody for treating chemotherapy-induced thrombocytopenia
Source: BMC Cancer. 2023 May 31;23:490. doi: 10.1186/s12885-023-10975-3 (PMC10230746; doi:10.1186/s12885-023-10975-3)
Supplement: Supplementary file 3 — Additional file 3: Supplementary Fig. 3. Effect of 2R13 on MK polyploidization inPB-CD34+ cells isolated from donor 2. [file 12885_2023_10975_MOESM3_ESM.pdf]

Supplementary Fig. 3 Effect of 2R13 on MK polyploidization in PB-CD34$^+$ cells isolated from donor 2

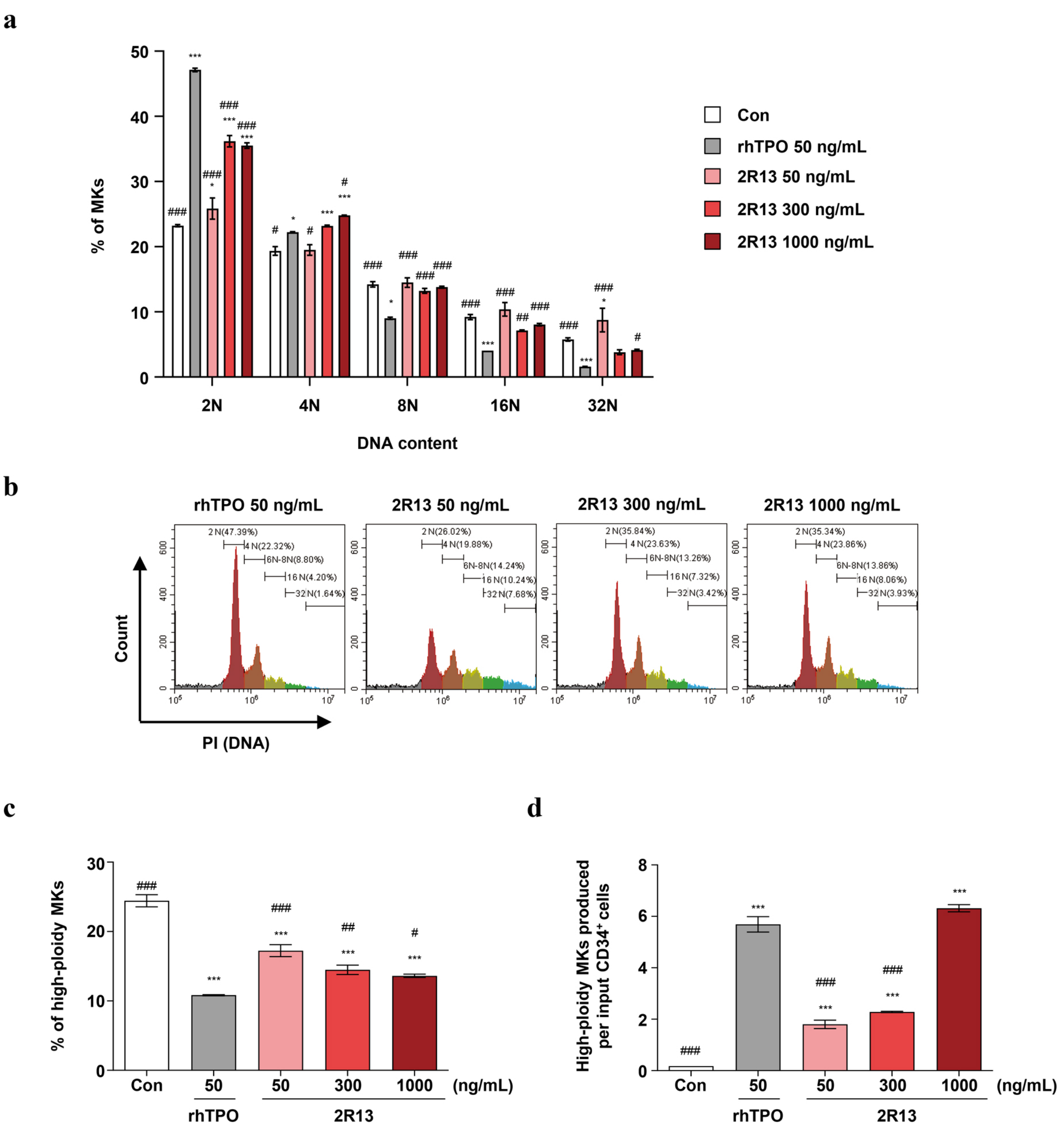

PB-CD34$^+$ cells derived from donor 2 were stimulated with rhTPO or 2R13 at the indicated concentrations for 13 days. **a** Ploidy status of CD41a$^+$ cells. **b** Representative flow cytometric profile of ploidy levels. **c, d** Percentage and number of high-ploidy (≥8N) MKs. The number of cells produced per input of PB-CD34$^+$ cells was calculated by multiplying the number of total nucleated cells with the percentage of high-ploidy CD41a$^+$ cells. Data are the mean ± SD ($n = 3$). One-way and two-way ANOVA were used for statistical analysis. *$p < 0.05$ and ***$p < 0.001$ vs control; #$p < 0.05$, ##$p < 0.01$, and ###$p < 0.001$ vs rhTPO.
