# Supplementary material for: Thrombopoietin receptor agonist antibody for treating chemotherapy-induced thrombocytopenia
Source: BMC Cancer. 2023 May 31;23:490. doi: 10.1186/s12885-023-10975-3 (PMC10230746; doi:10.1186/s12885-023-10975-3)
Supplement: Supplementary file 5 — Additional file 5: Supplementary Fig. 5. MPL signal transduction induced by 2R13 inhuman platelets from donor 2 and donor 3. [file 12885_2023_10975_MOESM5_ESM.pdf]

Supplementary Fig. 5 MPL signal transduction induced by 2R13 in human platelets from donor 2 and donor 3

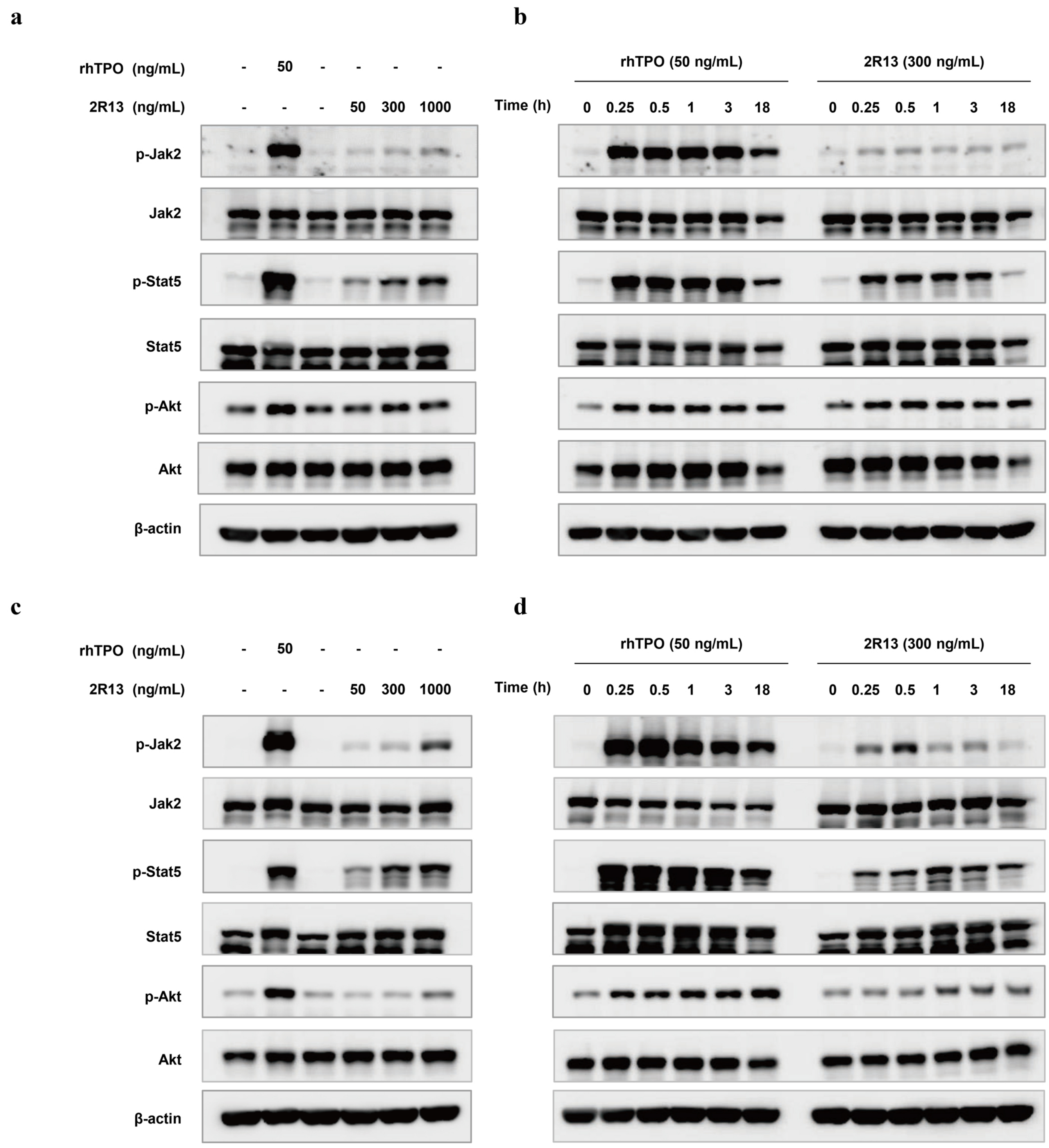

Western blot analysis of human platelets derived from donors 2 (**a, b**) and donor 3 (**c, d**), stimulated with rhTPO or 2R13, using the indicated antibodies. **a, c** rhTPO or 2R13 was added at the indicated concentrations for 30 min. **b, d** rhTPO (50 ng/mL) or 213 (300 ng/mL) was treated over the indicated period.
