# Supplementary material for: Thrombopoietin receptor agonist antibody for treating chemotherapy-induced thrombocytopenia
Source: BMC Cancer. 2023 May 31;23:490. doi: 10.1186/s12885-023-10975-3 (PMC10230746; doi:10.1186/s12885-023-10975-3)
Supplement: Supplementary file 6 — Additional file 6: Supplementary Fig. 6. Flow cytometric analysis of LSK+ cells. [file 12885_2023_10975_MOESM6_ESM.pdf]

Supplementary Fig. 6 Flow cytometric analysis of LSK$^+$ cells

a

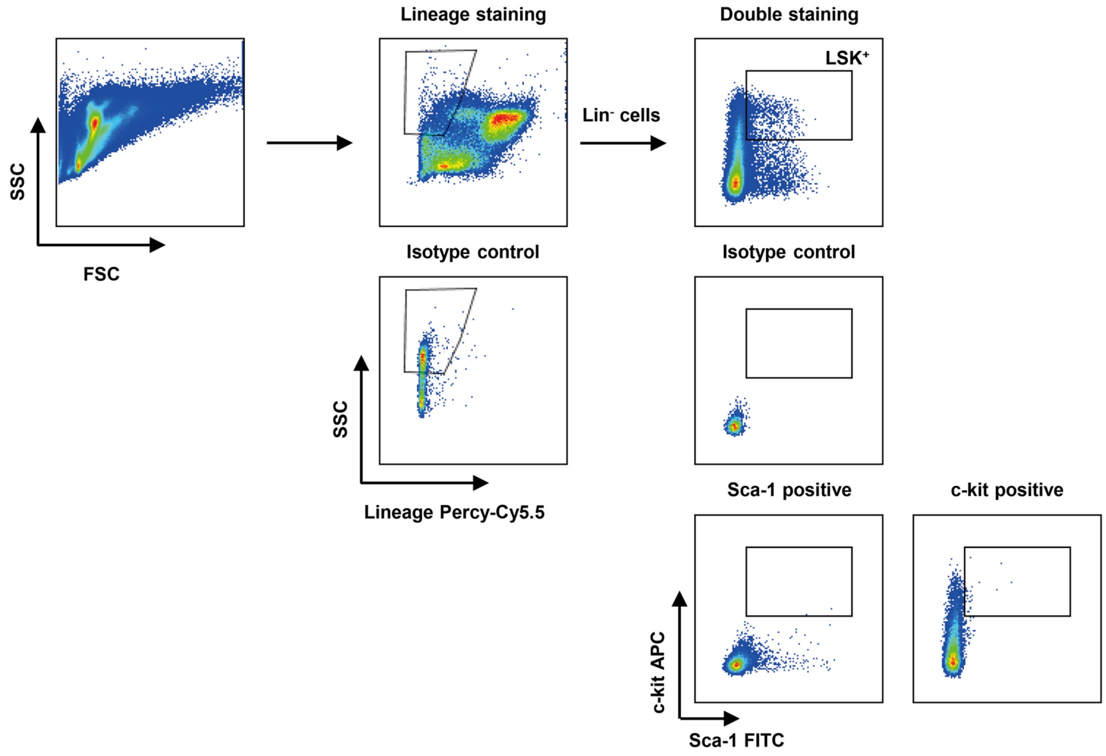

a Flow cytometry gating strategy used to isolate LSK$^+$ cells (Lin$^-$/Sca-1$^+$/c-kit$^+$) derived from BM.
