# Supplementary material for: Thrombopoietin receptor agonist antibody for treating chemotherapy-induced thrombocytopenia
Source: BMC Cancer. 2023 May 31;23:490. doi: 10.1186/s12885-023-10975-3 (PMC10230746; doi:10.1186/s12885-023-10975-3)
Supplement: Supplementary file 8 — Additional file 8: Supplementary Fig. 8. Uncropped full-length images of westernblotting membranes. [file 12885_2023_10975_MOESM8_ESM.pdf]

Supplementary Fig.8 Uncropped full-length images of western blotting membranes

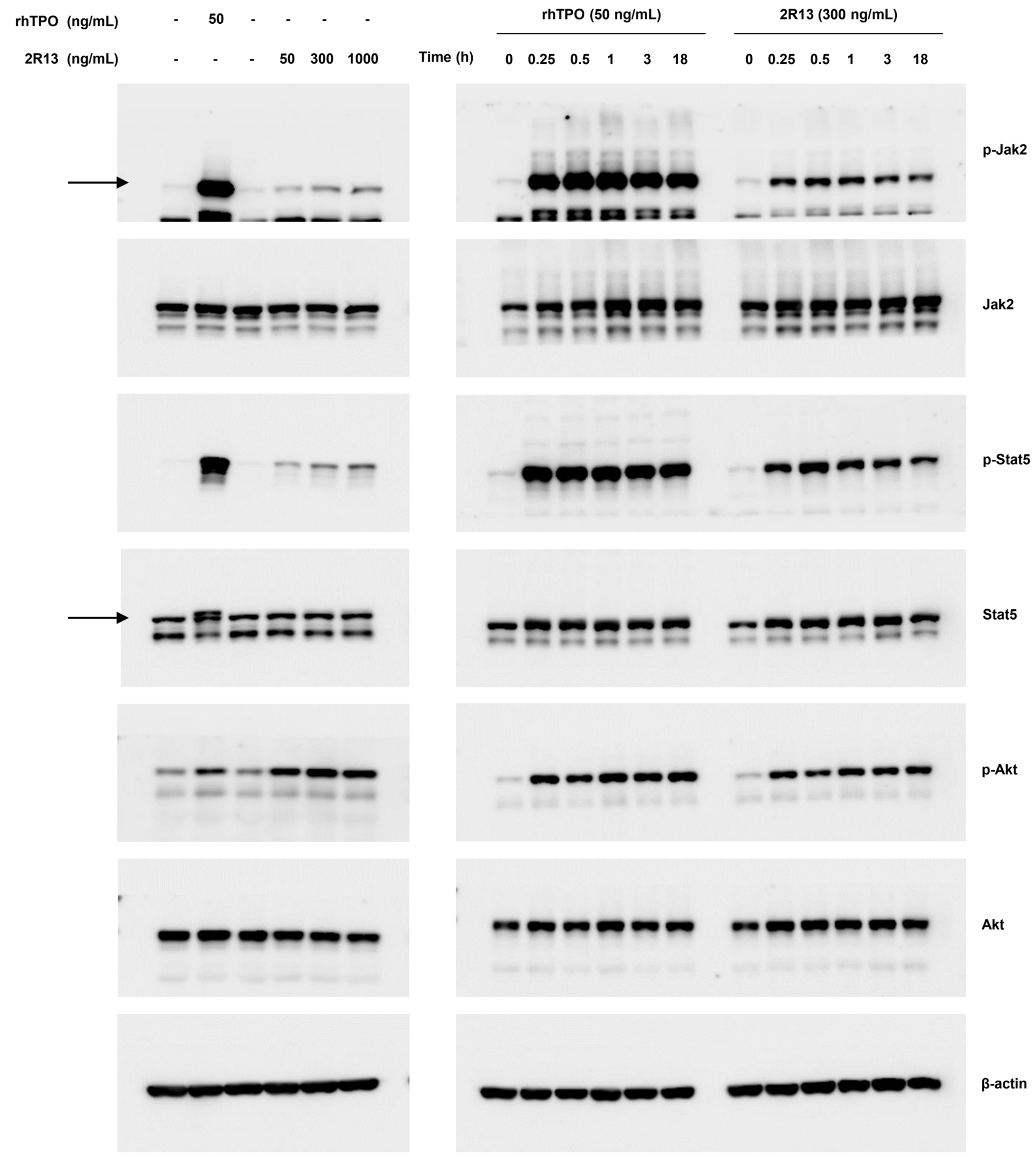

Uncropped full-length images of western blotting membranes presented in Fig. 5a and b. Membranes were cut to enable blotting for multiple antibodies, and there are no images of adequate length.
